# A Case Report of Subcutaneous Ossifying Fibromyxoid Tumour of the Back

**DOI:** 10.7759/cureus.62751

**Published:** 2024-06-20

**Authors:** Ting Fong Yeo, Caitlin Borowsky, Wael Hamarneh, Kazeem Salako

**Affiliations:** 1 Dermatology, Northampton General Hospital, Northampton, GBR; 2 Histopathology and Cytopathology, Northampton General Hospital, Northampton, GBR

**Keywords:** rare tumors, dermatology case report, histology and histopathology, ossifying fibromyxoid tumour, subcutaneous tumor

## Abstract

Ossifying fibromyxoid tumour (OFMT) is a rare subcutaneous soft tissue neoplasm, with unclear lineage and intermediate differentiation. Typically presenting as a benign growth, however it can recur locally, and malignant variants have been reported. We present an unusual case of OFMT occurring as a subcutaneous mass on the right upper back.

A 29-year-old gentleman presented with one-year history of a painless, slowly enlarging mass on his right upper back. He had no relevant medical history, was not on any medications, and had no family history of skin cancer. Physical examination revealed a 25mm x 25mm skin-coloured, stony-hard, well circumscribed mass. The initial clinical diagnosis was a calcified epidermoid cyst. The lesion was excised and sent for histopathology, which revealed well-circumscribed cellular spindle cells with low mitotic index. Immunohistochemistry showed variable S100 positivity. Due to its rarity, the diagnosis was delayed as the biopsy required a second opinion from a tertiary hospital. The final pathological diagnosis confirmed OFMT.

This case describes the very rare presentation of OFMT in a dermatology clinic, highlighting the importance of recognising this neoplasm due to its potential for recurrence and metastasis. This unusual case of OFMT broadens the dermatological differential diagnosis for a subcutaneous mass.

## Introduction

Ossifying fibromyxoid tumour (OFMT) is a rare mesenchymal soft tissue neoplasm with intermediate differentiation and unclear lineage [1]. The causes and mechanisms of differentiation for OFMT remain unknown. It primarily occurs in the deep soft tissue of the trunk and proximal extremities, affecting men more frequently than women, with a median age of 50 years. Although typically benign, cases of local recurrence and metastasis have been reported. Accurate diagnosis of OFMT is challenging due to its rarity and the variability in its histology and immunohistochemical features [2].

## Case presentation

**Figure 1:**
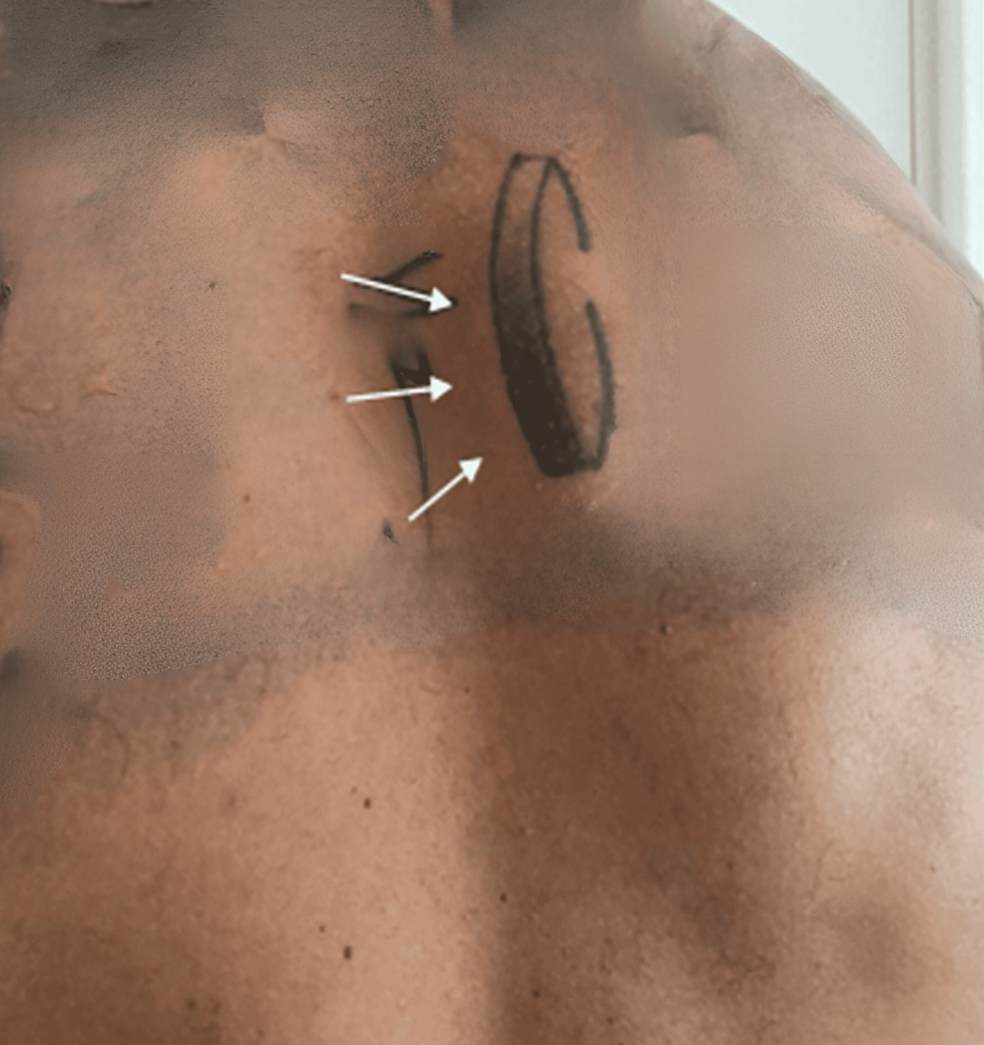
Clinical image demonstrating a 25mm x 25mm subcutaneous nodule (white arrows).

A 29-year-old man presented to our dermatology clinic with a one-year history of a painless, slowly growing mass on his right upper back. Physical examination revealed a 25mm x 25mm stony-hard, skin-coloured non-tender mass (Figure 1). No contributory past medical problems, medications or family history of skin cancer was reported.

**Figure 2:**
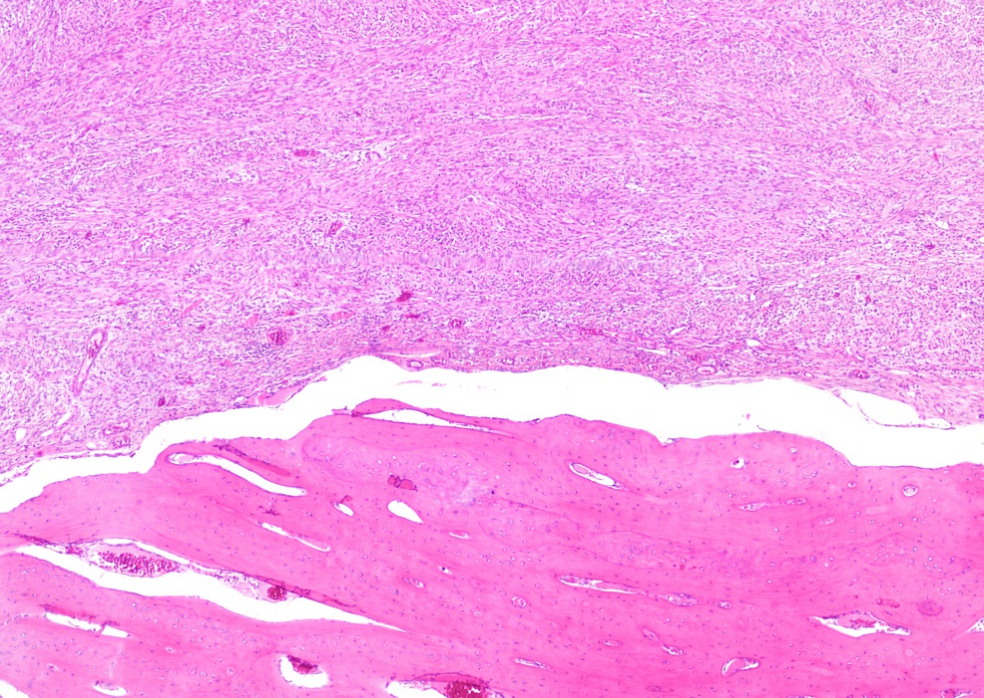
Interface between mature bone and spindle cell component

**Figure 3:**
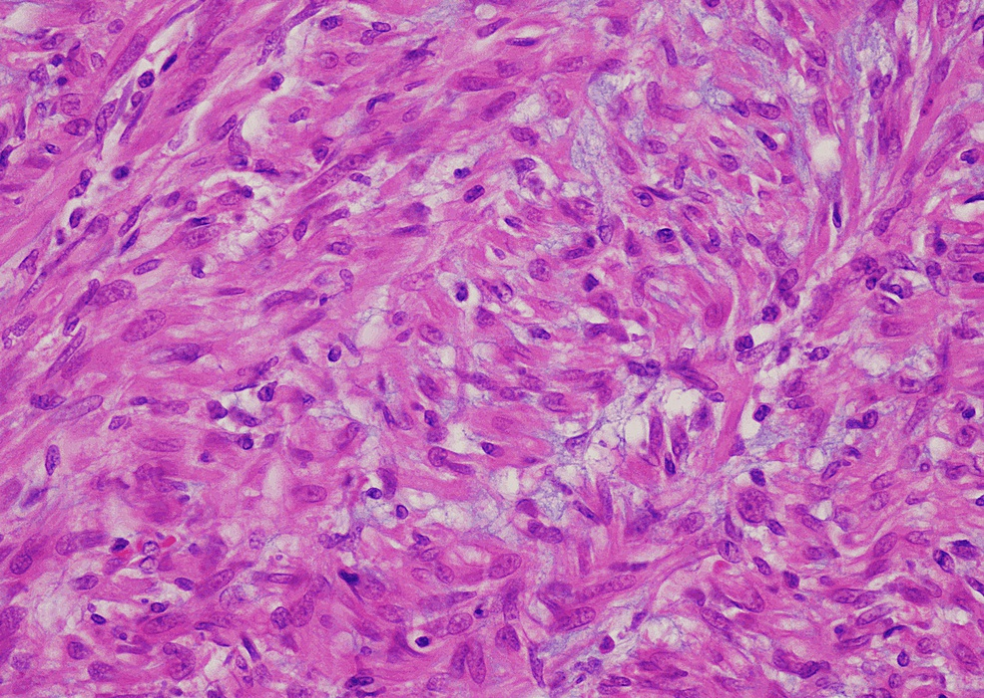
Spindle cell component of the lesion showing bland spindle cells and fibromyxoid stroma.

The histopathological examination of the mass revealed a well-circumscribed spindle cell tumour with an incomplete cuff of bone (Figure 2). The spindle cell lesions displayed variable cellularity, featuring areas of hypocellular myxoid stroma (Figure 3) and other areas with more cellular spindle cell proliferation. These spindle cells appeared plump with eosinophilic cytoplasm, forming nests and cords in a slightly myxoid stroma. The mitotic index was low, and no malignant cytological features were observed.

**Figure 4:**
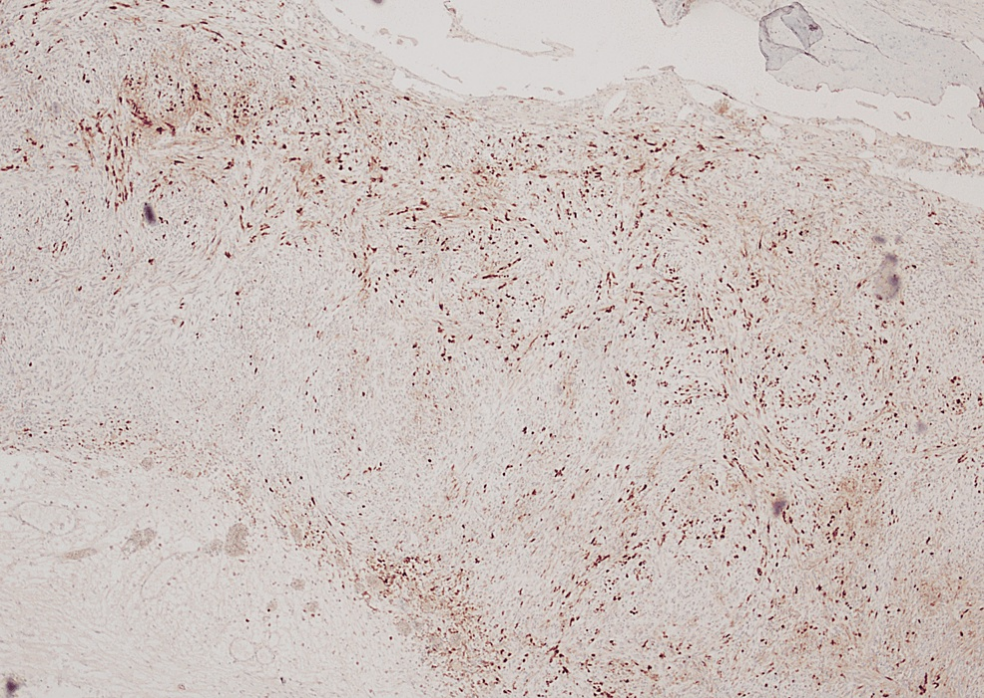
Patchy positivity of spindle cells for S100 protein

**Figure 5:**
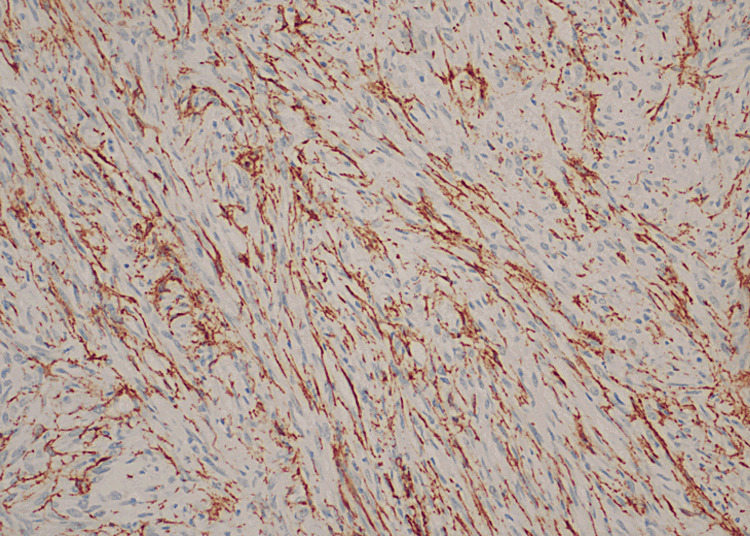
Vessels highlighted by SMA. SMA: smooth muscle actin

**Figure 6:**
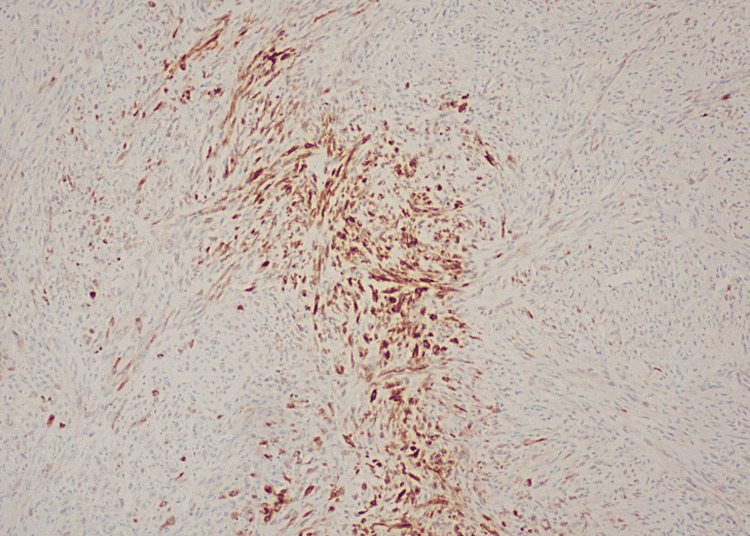
Some focal areas with EMA positivity EMA: epithelial membrane antigen

**Figure 7:**
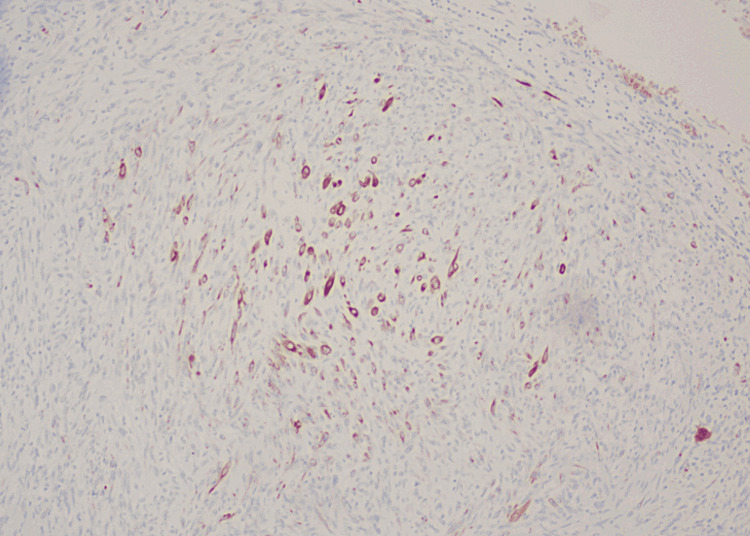
MNF116 showed occasional positive spindle cells.

Immunohistochemistry analysis showed patchy S100 positivity in spindle cells (Figure 4), while smooth muscle actin (SMA) highlighted the vessels (Figure 5). Epithelial membrane antigen (EMA) exhibited positivity only in a focal area (Figure 6) with the majority of the tumour cells showing negativity. MNF116 showed occasional positive spindle cells (Figure 7). The Ki-67 proliferation index was notably low and SOX-10 was negative. Morphologically, no malignant cytological features were observed. The local excision was comprehensive.

Due to the extreme rarity of this soft tissue tumour, the histopathology slides were sent to a tertiary centre for further evaluation to confirm the diagnosis. The final histopathology report confirmed the diagnosis of OFMT. The patient has remained well following the complete excision of the lesion, with no recurrence reported for over two years.

## Discussion

OFMT is a rare type of soft tissue neoplasm with ambiguous differentiation, first described by Enzinger et al. in 1989 [3]. While some evidence suggests OFMTs come from cartilaginous, smooth muscle, Schwannian or neuronal differentiation, this has not been conclusively proven [4]. Typically, it arises in the subcutaneous tissues of the extremities, or trunk and predominantly affects middle-aged men. Our patient presented atypically at the younger age of 29.

Clinically, OFMT presents as a painless, well-demarcated, non-tender soft tissue tumour, ranging in size from 1cm to 14cm, with an average size of 5cm [5]. Their consistency can vary from lobulated and firm to cystic, rubbery or stony-hard [5]. Macroscopically, OFMTs are characterized by a fibrous thick pseudo capsule surrounded by an incomplete peripheral layer of lamellar bone. Microscopically, OMFTs consist of uniform, round spindle-shaped, ovoid cells organised in cords, clusters, sheets or nests in a fibromyxoid matrix. Mitotic figures may be observed, whilst necrosis and vascular invasion are uncommon [6].

Immunohistochemically, S-100 and desmin serve as useful adjunct staining markers for OMFTs, identified in approximately 75% and 25% of cases, respectively [4]. Co-expression of markers such as vimentin, desmin, EMA, SMA, EAAT4, MUC4, NFP and CD56 has been reported in certain cases [7]. Malignant OFMTs are less frequently positive for S-100 and desmin, and more frequently positive for pancytokeratin, EMA and actin [5]. Recent studies have identified specific molecular translocations that underlie OFMTs, enhancing diagnosis through molecular and genetic features [5]. PHF1 gene arrangement has been observed in 80% of cases, including benign, atypical and malignant subtypes, with fusion to EP400 in 44% of these cases [5]. ZC3H7B-BCOR and MEAF6-PHF1 fusions are predominantly found in S-100 negative and malignant OFMT [5]. Our case did not exhibit histological features of the malignant variant.

Accurate histopathologic diagnosis of OFMT is essential to distinguish this tumour from its differential diagnoses, such as epithelioid schwannoma and soft tissue myoepithelioma which also share similar immunological and cytoarchitectural features [1]. Mixed tumours/myoepitheliomas do not have surrounding bone and exhibit positive staining for cytokeratins, which are absent in OFMT [1]. A panel including keratin AE1/AE3, EMA, S100 and glial fibrillary acidic protein (GFAP) can identify myoepithelial differentiation in most cases [8]. Epithelioid schwannomas usually arise near a nerve, a feature uncommon in OFMT cases [1]. Immunohistochemical features indicative of epithelioid schwannoma include diffuse immunoreactivity for SOX10 or the presence of EMA or GLUT1+ perineural cells within the fibrous capsule [8]. In our case, a delay in diagnosis occurred due to rarity and histological complexity. The biopsy was sent to a tertiary centre for further review. This underscores the need for greater awareness and expertise in recognising and managing OFMTs.

There are three variants of OFMTs: benign (typical), malignant and atypical. Benign OFMTs are the most common, histologically characterized by low nuclear grade, high cellularity and a mitotic rate of less than 2 per 50 high-power fields (HPF). Malignant OFMTs are very rare and exhibit high nuclear grade, high cellularity and a mitotic activity greater than 2 per 50 HPF [4]. The atypical variant includes lesions with atypical features that do not fully meet the criteria for malignant or typical subtypes. Miettinen et al. [2] reported an increased mitotic activity as a risk factor for local recurrences, while Folpe et al. [9] described the malignant variant as prone to local recurrence and metastasis.

Although OFMTs are rare and usually benign, they do have metastatic potential and can exhibit aggressive features even in typical variants, including the possibility of local recurrence after surgical removal [9]. In rare instances, OFMTs may metastasise, with a 10% mortality rate in patients with metastatic disease [9], usually spreading to the lungs and soft tissue [4]. The primary treatment is complete surgical removal, followed by regular surveillance postoperatively.

## Conclusions

We present a very rare case of a typical variant of OFMT that appeared as a subcutaneous mass in a dermatology clinic. OFMTs are mesenchymal neoplasms of unknown origins, exhibiting variable histological features that can resemble other soft tissue tumours, making accurate identification challenging. This case highlights the importance of maintaining strong clinical suspicion and utilising all available diagnostic tools to accurately diagnose OFMTs. Considering its unpredictable potential for recurrence and metastasis, early recognition is crucial. Close, long-term postoperative follow-up is essential to monitor for local recurrence and distant metastases, particularly in atypical and malignant types. More studies are needed to better understand the pathogenesis of OFMTs and to determine the optimal treatment.
